# From social and emotional learning to mathematics engagement: the mediating role of mathematical resilience

**DOI:** 10.3389/fpsyg.2026.1827619

**Published:** 2026-10-12

**Authors:** Melek Alemdar, Mahmut Sami Yiǧiter, Betül Gever

**Affiliations:** 1 Ahmet Keleşoglu Education Faculty, Necmettin Erbakan University, Konya, Türkiye; 2 Manchester Institute of Education, The University of Manchester, Manchester, United Kingdom; 3 Distance Education Application and Research Center, Social Sciences University of Ankara, Ankara, Türkiye; 4 University of Salford, Manchester, United Kingdom; 5 National Ministry of Education, Konya, Türkiye

**Keywords:** mathematical resilience, mathematics engagement, mediation model, SEL, social and emotional learning, structural equation modeling, Türkiye

## Abstract

**Introduction:**

Mathematics engagement is often conceptualized primarily as a cognitive or instructional outcome. However, emerging research suggests that students' engagement in mathematics may also depend on social and emotional learning (SEL) competencies and their capacity to cope with academic challenge. This study examined whether SEL competencies contribute to middle school students' mathematics engagement both directly and indirectly through mathematical resilience.

**Method:**

In this cross-sectional correlational study, data were collected from 373 middle school students in Konya, Türkiye, recruited through convenience sampling. A structural equation model was estimated to examine whether SEL competencies predict behavioral, cognitive, emotional, and social dimensions of mathematics engagement directly and indirectly through mathematical resilience.

**Results:**

SEL significantly predicted mathematical resilience (β = 0.58, *p* < 0.001), and mathematical resilience strongly predicted mathematics engagement (β = 0.60, *p* < 0.001). SEL also retained a significant direct effect on engagement (β = 0.33, *p* < 0.001), while the indirect effect through mathematical resilience was significant, indicating partial mediation.

**Conclusion:**

The findings suggest a dual pathway through which SEL competencies support mathematics engagement: by fostering resilience in response to mathematical difficulty and by directly supporting students' participation in classroom learning processes. These results highlight the importance of integrating SEL within mathematics education to support sustained engagement with mathematics; however, findings should be interpreted considering the cross-sectional design and convenience sampling.

## 1 Introduction

Imagine a middle school mathematics classroom designed to foster collaborative activity, one in which students are expected to articulate their thinking, negotiate meaning and work through challenging ideas together. In a recent cooperative small-group instruction study we conducted in fifth-grade mathematics, we implemented the Student Teams-Achievement Divisions (STAD) model to support equitable participation (Güven and Alemdar, 2025). Although collaboration produced substantial gains in students' mathematics engagement, our classroom observations revealed a persistent tension: even when collaborative tasks were carefully structured, participation often remained uneven. Some students dominated the discussion, others withdrew, negotiation skills were fragile, and the teacher repeatedly intervened to regulate interaction. As Rimm-Kaufman et al. (2025) note, such breakdowns rarely reflect cognitive difficulty alone; rather, they signal insufficient development or enactment of the social and emotional competencies required for productive academic collaboration.

This pattern aligns with a broad consensus that mathematics learning is inherently affective and social. Emotional variables, including anxiety, self-efficacy, and motivational beliefs shape students' willingness to engage with mathematical content (Jain and Dowson, 2009; Ramirez et al., 2018; Robbins et al., 2004), as do peer interaction dynamics and relational climates (Semeraro et al., 2020). Fixed-ability beliefs and competitive environments depress engagement (Szczygieł and Pieronkiewicz, 2022), whereas supportive relationships promote risk-taking and persistence. Emotional experiences during mathematical work are systematically associated with performance, positioning emotion regulation as a key process linking engagement and achievement (Paik et al., 2025). These findings suggest that the affective and social demands embedded in mathematics learning are not peripheral but structurally integral to students' academic functioning. Engaging in mathematical activity requires learners to regulate emotion during uncertainty, sustain motivation amid difficulty, collaborate productively, and seek support strategically—capacities centrally reflected in contemporary models of social and emotional learning (SEL; CASEL, 2025; Wigelsworth et al., 2024).

Guided by the integrative framework of motivation (Skinner et al., 2008), engagement can be understood as the outward manifestation of motivational processes shaped by students' perceptions of competence, autonomy, and relatedness. Engagement emerges when learners experience themselves as capable, supported, and agentic; disengagement arises when these self-systems are undermined. Building on this perspective, Wang et al. (2016) conceptualized engagement in mathematics classrooms as comprising behavioral, emotional, cognitive, and social dimensions. Because mathematics learning places particular demands on these self-systems, requiring students to interpret challenge adaptively, regulate affect, and navigate peer interactions (Rimm-Kaufman et al., 2015), the competencies reflected in SEL become theoretically central to sustaining engagement in mathematics.

Within this theoretical framing, mathematical resilience represents a domain-specific adaptive mechanism through which SEL competencies may translate into sustained mathematics engagement. Mathematical resilience reflects a positive adaptive stance that enables learners to continue working despite obstacles (Johnston-Wilder and Lee, 2010). Beyond persistence, it involves valuing mathematics, maintaining growth-oriented beliefs, employing effective strategies, and recognizing available support (Lee and Johnston-Wilder, 2017). Empirical research links mathematical resilience with higher achievement, stronger motivation, and greater participation in mathematical discourse (Öztürk and Kilmen, 2022; Marangoz, 2024). Classrooms that normalize mistakes and foster psychological safety appear especially conducive to its development (Akkan and Horzum, 2024; Reyes et al., 2012).

The Turkish educational context further underscores the relevance of examining these associations. The Ministry of National Education's K12 Skills Framework Türkiye Integrated Model reconceptualizes mathematical competence as an integration of disciplinary skills, SEL competencies, and learner dispositions [Ministry of National Education (MEB), 2023, 2024; Karabey and Erdogan, 2023]. By embedding SEL within mathematics-specific learning processes, the framework explicitly positions socio-emotional capacities and adaptive dispositions as foundational to productive mathematical engagement. Empirically examining SEL, mathematical resilience, and engagement within this context, therefore, provides timely evidence aligned with ongoing curricular reforms.

Developmentally, early adolescence (approximately ages 11–14) represents a particularly important period in which to investigate these associations. Students experience heightened sensitivity to social evaluation, expanding peer networks, and increased metacognitive capacity (Eccles, 1999). Social–emotional competencies such as self-regulation and adaptive coping consolidate during this stage (Murray et al., 2015), while academic expectations intensify as students transition toward more demanding educational contexts (Panayiotou and Humphrey, 2018). These converging developmental pressures magnify the emotional and interpersonal demands of mathematics learning, making it theoretically plausible that SEL competencies influence engagement through adaptive mechanisms such as resilience.

Although meta-analytic evidence indicates consistent positive associations between SEL programs and academic outcomes (Durlak et al., 2011; Cipriano et al., 2023), variability across subject domains underscores the need to identify proximal explanatory pathways (Lemberger et al., 2015; Bakosh et al., 2016). Recent synthesized evidence indicates that SEL programs yield substantial improvements in mathematics achievement (47.5%) despite not teaching mathematical content directly (Ha et al., 2025). Such findings suggest that SEL's academic effects may operate through motivational, emotional, and engagement-related mechanisms. However, empirical studies directly examining the integrated functioning of SEL, mathematical resilience, and mathematics engagement—particularly among middle school students—remain limited.

The present study addresses two interrelated gaps in the literature. First, middle school populations remain underrepresented in empirical work on SEL and academic processes, despite the heightened developmental salience of early adolescence (Ha and Cipriano, 2025; Domitrovich et al., 2022). Second, no prior study has empirically tested a dual-pathway model, comprising both direct and resilience-mediated effects of SEL on mathematics engagement, among middle school students. To our knowledge, this is the first such study. The current study therefore tests whether SEL competencies predict mathematics engagement directly and indirectly through mathematical resilience, within the Turkish middle school context, thereby generating evidence aligned with ongoing national curricular reforms. Grounded in the theoretical and empirical literature reviewed above, the present study proposes the following hypotheses:

**H1**. *Social and emotional learning (SEL) competencies will be positively associated with students' mathematical resilience*.

Students who demonstrate stronger SEL capacities are expected to show higher levels of mathematical resilience.

**H2**. *Mathematical resilience will be positively associated with mathematics engagement*.

Students with higher resilience are expected to exhibit stronger behavioral, cognitive, emotional, and social engagement in mathematics learning.

**H3**. *SEL competencies will be positively associated with mathematics engagement*.

Consistent with prior research, students with stronger SEL capacities are expected to demonstrate higher engagement in mathematics classrooms.

**H4 (Mediation Hypothesis)**. *Mathematical resilience will mediate the relationship between SEL competencies and mathematics engagement*.

Specifically, SEL is expected to enhance students' engagement indirectly by strengthening their mathematical resilience, which functions as a proximal socio-cognitive mechanism supporting sustained involvement in mathematical activity.

## 2 Method

### 2.1 Research design and model

This study employed a cross-sectional quantitative, correlational design to examine the associations among social and emotional learning (SEL), mathematical resilience, and mathematics engagement in middle school students. Correlational designs are well suited for investigating naturally occurring relationships among psychological and educational constructs without manipulating variables or introducing experimental conditions (Creswell, 2014). Consistent with this approach, no instructional intervention was implemented; rather, the study aimed to model the structural relations among the variables as they exist within authentic school environments.

Guided by the hypothesized conceptual framework (Figure 1), SEL competencies were specified as the exogenous predictor, mathematical resilience as the mediator, and mathematics engagement as the endogenous outcome variable. This design enabled the examination of both direct associations (e.g., SEL → engagement) and indirect pathways (SEL → mathematical resilience → engagement), consistent with contemporary mediation models used in educational psychology.

**Figure 1:**
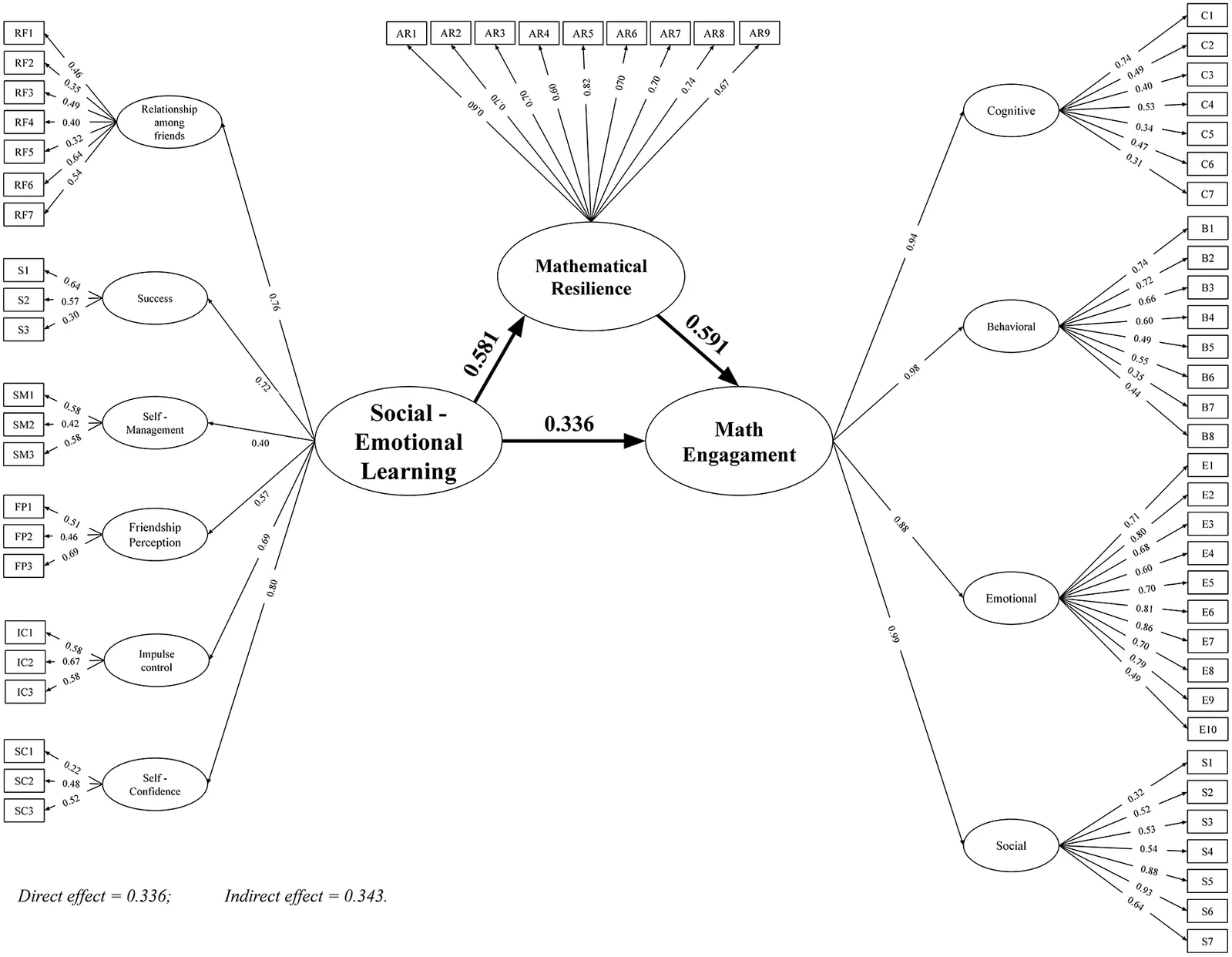
Structural equation model testing the mediating role of mathematical resilience (MR) in the association between social and emotional learning (SEL) and mathematics engagement (ME). Standardized path coefficients are shown on structural paths.

The analytic focus, therefore, was on testing whether mathematical resilience statistically mediates the association between SEL competencies and students' behavioral, cognitive, emotional, and social engagement in mathematics learning.

### 2.2 Participants

In the spring term of the 2025–2026 academic year, middle school students from three public schools in Konya, Turkey, participated in the study. The initial sample comprised 419 students (See Table 1). A convenience sampling strategy was employed due to practical constraints related to time, accessibility, and available resources. Access to schools was contingent upon administrative permissions, school willingness to participate, and feasibility within the data collection timeline. Therefore, schools that granted access during the spring semester and represented typical public middle school settings in Konya were included in the study. Following data screening procedures (see Data Analysis), cases displaying inconsistent response patterns were excluded, yielding a final analytic sample of 373 students.

**Table 1:** Demographic characteristics of the participants.

Variable	Category	Frequency	Percentage
Grade	5th grade	109	29.22
	6th grade	119	31.90
	7th grade	79	21.18
	8th grade	66	17.69
Mother's graduation	Illiterate	24	6.43
	Primary school	102	27.35
	Middle school	118	31.64
	High school	87	23.32
	University	31	8.31
	Master's degree	11	2.95
Father's graduation	Illiterate	12	3.22
	Primary school	79	21.18
	Middle school	113	30.29
	High school	122	32.71
	University	31	8.31
	Master's degree	16	4.29
Family income	Low income	30	8.04
	Lower-middle income	95	25.47
	Upper-middle income	131	35.12
	High income	117	31.37

As presented in Table 1, the sample included students from all middle school grade levels, with the largest proportion enrolled in 6th grade (31.90%), followed by 5th grade (29.22%), 7th grade (21.18%), and 8th grade (17.69%), indicating a relatively balanced distribution across grade levels. Parental education levels were concentrated primarily at the middle school and high school levels for both mothers and fathers. Specifically, mothers' education was most frequently reported as middle school (31.64%), whereas fathers' education was most commonly high school (32.71%). In terms of household income, the majority of students were from upper-middle income (35.12%) and high income (31.37%) households, suggesting that the sample largely represented students from middle-to-higher socioeconomic backgrounds.

### 2.3 Measures

#### 2.3.1 Social-emotional learning skills scale

The social-emotional learning skills scale was developed by Esen-Aygün and Sahin-Taşkin (2017) to assess students' social–emotional competencies. The scale consists of 27 items rated on a 3-point Likert scale (1 = Never, 2 = Sometimes, 3 = Always). It captures seven dimensions named relationship among friends, friendship perception, success, self-management, impulse control, self-confidence, and persistence. Higher scores indicate higher SEL skills.

Psychometric evidence from the original validation study (*N* = 439) demonstrated robust structural validity. Exploratory factor analysis supported a seven-factor structure explaining 51.9% of the total variance, with strong sampling adequacy (KMO = 0.872; Bartlett's test *p* < 0.001). Confirmatory factor analysis indicated excellent fit (χ^2^/df = 1.65, RMSEA = 0.036, CFI = 0.97, GFI = 0.93). Internal consistency was high for the overall scale (α = 0.86). In the present study, the full 27-item scale was administered to all participants. During CFA, the final retained measurement model included fewer items because several indicators and the SEL-Persistence dimension were removed on psychometric grounds. Thus, the reference to the 21-item structure reflects CFA-based model revision rather than an *a priori* shortening of the scale before data collection. Reliability estimates for the retained SEL measurement model were modest but within the lower bound of acceptability (Cronbach's α = 0.65, McDonald's ω = 0.65).

#### 2.3.2 Math and science engagement scale

Mathematics engagement was measured using the scale originally developed by Wang et al. (2016) and later adapted into Turkish by Turan Gürbüz et al. (2020). The instrument consists of 33 items assessing four engagement dimensions, cognitive, behavioral, emotional, and social engagement, rated on a 5-point Likert scale ranging from 1 (strongly disagree) to 5 (strongly agree). Higher scores indicate stronger engagement in mathematics.

The Turkish adaptation demonstrated strong psychometric qualities, with prior studies reporting high internal consistency for the overall scale (α ≈ 0.90) and acceptable split-half reliability (e.g., Guttman coefficients around 0.80). Confirmatory factor analysis in the adaptation study supported the original four-factor structure with excellent model fit indices (e.g., χ^2^/df = 1.75; RMSEA = 0.038; CFI = 0.98; NFI = 0.96). In the present study, the full 33-item scale was administered. During CFA, one item was removed due to a standardized factor loading below 0.30; therefore, the final retained measurement model included 32 items. The reliability values of the retained scale were high (Cronbach's α = 0.91, McDonald's ω = 0.93).

#### 2.3.3 Academic resilience in mathematics scale

Mathematical resilience was assessed using the Academic Resilience in Mathematics Scale (ARMS) originally developed by Ricketts et al. (2015). The scale consists of 9 items rated on a 6-point Likert scale ranging from 1 (strongly disagree) to 6 (strongly agree), with higher scores indicating greater resilience in mathematics learning. The original instrument has demonstrated strong psychometric properties, including acceptable internal consistency (α ≈ 0.79) and item fit indices obtained through Many-Facet Rasch modeling.

The Turkish adaptation of the scale was validated by Pekdemir et al. (2019) with a large sample of 930 seventh- and eighth-grade students. Exploratory factor analysis supported a unidimensional structure explaining 49.41% of the variance, and confirmatory factor analysis indicated good model fit (χ^2^/df = 2.71; GFI = 0.97; AGFI = 0.94; CFI = 0.98; RMSEA = 0.06; SRMR = 0.04). Reliability analyses showed high internal consistency (α = 0.86–0.87 across samples) and strong test–retest stability (*r* = 0.80). Criterion-related validity was supported through expected correlations with mathematics anxiety (negative) and academic self-efficacy (positive). In our study, all nine items were administered, and the scale's measurement structure was further evaluated through confirmatory factor analysis (see Measurement Model section). The reliability values of the scale were high (Cronbach Alpha α = 0.83, McDonald Omega ω = 0.87).

### 2.4 Data analysis

A total of 419 students initially participated in the study. Prior to the main analyses, data screening procedures were conducted to identify missing responses, aberrant response patterns, violations of normality, and multicollinearity. No missing values were detected in the observed indicators. Aberrant response patterns were examined using the Lz person-fit index (Drasgow et al., 1985), with a cut-off of |Lz| > 2.0 applied to identify inconsistent responding. Based on this criterion, 46 cases were flagged and removed listwise, resulting in a final analytic sample of 373 students. A comparison of descriptive statistics and model results before and after data screening indicated no meaningful differences.

A *post-hoc* power analysis based on RMSEA indicated that the present model achieved excellent statistical power (> 0.999) given the sample size (*N* = 373) and model degrees of freedom (df = 1,877), with α = 0.05 and RMSEA = 0.05. These results suggest that the sample size was more than sufficient to detect potential model misspecification at the global model level. However, this index reflects overall model fit sensitivity rather than power for specific structural paths in the mediation model.

Normality assumptions were evaluated using skewness and kurtosis values. Following commonly used guidelines (e.g., skewness within ± 2 and kurtosis within ± 7, with more liberal thresholds of ± 3 and ± 10; Kline, 2016), all variables demonstrated acceptable univariate normality. Multicollinearity was examined using Variance Inflation Factor (VIF) values derived from multiple regression analyses. VIF values ranged from 1.22 to 2.36, well below the commonly accepted cutoff of 5, indicating no multicollinearity concerns.

Following data screening, we evaluated the measurement properties and structural relations among the constructs using Confirmatory Factor Analysis (CFA) and Structural Equation Modeling (SEM). SEM allows for the simultaneous estimation of direct and indirect effects among latent variables while accounting for measurement error. Model fit was assessed using multiple indices, including the chi-square statistic (χ^2^), χ^2^/df ratio, Comparative Fit Index (CFI), Tucker–Lewis Index (TLI), Root Mean Square Error of Approximation (RMSEA), and Standardized Root Mean Square Residual (SRMR). Consistent with established criteria, χ^2^/df values < 3 indicate acceptable fit, CFI and TLI ≥ 0.90 indicate acceptable fit (≥ 0.95 good fit), RMSEA ≤ 0.06 indicates good fit ( ≤ 0.08 acceptable), and SRMR < 0.08 indicates good fit.

During CFA, standardized factor loadings were examined for all items. In line with recommendations in the measurement literature (Hair et al., 2010; Kline, 2016), items with loadings below 0.30 were considered weak indicators of their respective latent constructs. Three items—SEL2, SEL24, and ME8—exhibited factor loadings below this threshold. These items were removed not for the purpose of improving model fit but to strengthen the conceptual coherence and structural validity of the measurement model. Item removal decisions were based on a combined evaluation of theoretical justification and empirical criteria (Brown, 2015). Also, in the initial measurement model, a Heywood case was detected for the SEL-Persistence dimension. Specifically, the residual variance associated with this dimension was estimated as negative, and warnings indicated that the variance–covariance matrix was not positive definite. Such results indicate inadmissible parameter estimates and potential model misspecification (Kline, 2016; Brown, 2015). Therefore, to ensure stable and interpretable parameter estimates and to maintain a statistically acceptable measurement model, the SEL-Persistence dimension was removed from the analysis.

All statistical analyses were conducted in *R*. CFAs and SEM analyses were performed using the *lavaan* package. Person-fit analyses were carried out using the *PerFit* package. Principal component analyses were run using the psych package, and descriptive statistics were obtained with the *moments* package.

### 2.5 Ethics statement

This study was reviewed and approved by the Necmettin Erbakan University Social and Human Sciences Scientific Research Ethics Committee (Approval Date: 09 January 2026; Meeting No. 01; Decision No. 2026/02).

Permission to conduct the study was obtained from the relevant educational authorities and participating school administrations. The research involved the administration of anonymous questionnaires during regular school hours and posed minimal risk to participants. Students were informed about the purpose of the study, the voluntary nature of participation, and their right to decline or discontinue participation at any time without any academic consequences.

No personally identifiable information was collected. Data were anonymized at the point of collection and were used solely for scientific purposes in accordance with national regulations governing research in educational settings.

## 3 Results

### 3.1 Descriptive statistics

Descriptive statistics (means, standard deviations, skewness, and kurtosis) for the study variables are reported in Table 2. These statistics were used to characterize distributional properties and to evaluate normality assumptions prior to SEM. In addition, Pearson correlation coefficients were computed to examine the direction and strength of bivariate associations among the constructs.

**Table 2:** Descriptive statistics.

Subscale (Scale)	1	2	3	4	5	6	7	8	9	7	11	12	13	Mean	SD	Skew	Kurt
Relationship among friends (SEL)	1													19.906	2.169	−0.588	0.382
Success (SEL)	0.252	1												7.791	1.143	−1.139	1.548
Self-management (SEL)	0.253	0.144	1											7.946	1.170	−1.133	0.896
Friendship perception (SEL)	0.409	0.180	0.223	1										7.316	1.252	−0.693	0.221
Impulse control (SEL)	0.320	0.075	0.151	0.151	1									7.115	1.598	−0.716	−0.281
Self-confidence (SEL)	0.111	0.251	0.035	0.082	0.131	1								8.408	1.517	0.082	−0.296
Cognitive (AR)	0.301	0.246	0.129	0.142	0.272	0.187	1							28.544	4.867	−0.199	−0.110
Behavioral (AR)	0.288	0.264	0.152	0.227	0.320	0.253	0.638	1						30.448	5.501	−0.288	−0.505
Emotional (AR)	0.221	0.204	0.071	0.171	0.252	0.274	0.535	0.688	1					38.381	8.233	−0.549	−0.232
Social (AR)	0.298	0.241	0.118	0.219	0.310	0.238	0.55	0.692	0.670	1				62.850	10.821	−0.876	0.424
Social—emotional learning (SEL)	0.765	0.510	0.474	0.569	0.565	0.466	0.397	0.459	0.371	0.443	1			124.279	11.008	−0.394	0.091
Math engagament (ME)	0.322	0.274	0.129	0.227	0.337	0.283	0.755	0.874	0.884	0.866	0.487	1		283.397	44.896	−0.435	−0.227
Academic resilience (AR)	0.234	0.215	0.119	0.171	0.258	0.211	0.484	0.585	0.553	0.922	0.373	0.732	1	37.048	7.336	−1.233	1.245

Table 2 shows that correlations among the study variables were generally positive. At the total-score level, SEL showed a small-to-moderate association with mathematical resilience (MR; *r* = 0.373) and a moderate positive association with mathematics engagement (ME; *r* = 0.487). In comparison, the association between mathematical resilience and mathematics engagement was comparatively stronger (MR with ME; *r* = 0.732). Overall, the correlation pattern is consistent with the proposed model, suggesting that SEL is related to both resilience and engagement, and that resilience is more strongly related to engagement. These findings provide preliminary support for testing the structural model in subsequent analyses.

### 3.2 Assessment of the measurement model

Prior to testing the structural paths, confirmatory factor analyses (CFA) were conducted to evaluate the measurement properties of the included instruments. Tables 3, 4 reports standardized factor loadings, standard errors, explained variance (*R*^2^), and model fit indices for the SEL scale, the mathematics engagement scale, and the mathematical resilience scale.

**Table 3:** The convergence validity of the measurement model.

Variable	Dimension	Item	Est	se	*P*	R2
Social-emotional learning skills scale	Relationship among friends	RF1	0.478	0.064	^***^	0.228
		RF2	0.462	0.072	^***^	0.213
		RF3	0.450	0.075	^***^	0.202
		RF4	0.331	0.064	^***^	0.110
		RF5	0.371	0.065	^***^	0.138
		RF6	0.595	0.064	^***^	0.354
		RF7	0.477	0.063	^***^	0.227
	Success	S1	0.557	0.079	^***^	0.310
		S2	0.557	0.076	^***^	0.311
		S3	0.430	0.080	^***^	0.185
	Self-management	SM1	0.612	0.101	^***^	0.375
		SM2	0.519	0.091	^***^	0.269
		SM3	0.503	0.098	^***^	0.253
	Friendship perception	FP1	0.456	0.065	^***^	0.208
		FP2	0.526	0.066	^***^	0.277
		FP3	0.696	0.069	^***^	0.485
	Impulse control	IC1	0.531	0.072	^***^	0.282
		IC2	0.837	0.076	^***^	0.701
		IC3	0.469	0.073	^***^	0.220
	Self-confidence	SC21	0.190	0.086	0.027	0.036
		SC22	0.348	0.085	^***^	0.121
		SC23	0.691	0.141	^***^	0.478
Math engagement scale	Cognitive	C1	0.753	0.034	^***^	0.567
		C2	0.478	0.045	^***^	0.228
		C3	0.381	0.049	^***^	0.145
		C4	0.507	0.045	^***^	0.257
		C5	0.370	0.051	^***^	0.137
		C6	0.467	0.049	^***^	0.218
		C7	0.324	0.053	^***^	0.105
	Behavioral	B1	0.791	0.023	^***^	0.626
		B2	0.717	0.030	^***^	0.515
		B3	0.645	0.035	^***^	0.416
		B4	0.584	0.040	^***^	0.341
		B5	0.454	0.043	^***^	0.206
		B6	0.545	0.041	^***^	0.297
		B7	0.362	0.048	^***^	0.131
		B8	0.452	0.046	^***^	0.204
	Emotional	E1	0.703	0.032	^***^	0.495
		E2	0.790	0.025	^***^	0.623
		E3	0.654	0.036	^***^	0.428
		E4	0.590	0.038	^***^	0.348
		E5	0.711	0.031	^***^	0.506
		E6	0.823	0.021	^***^	0.677
		E7	0.866	0.017	^***^	0.750
		E8	0.712	0.032	^***^	0.507
		E9	0.795	0.025	^***^	0.631
		E10	0.486	0.043	^***^	0.236
	Social	S1	0.245	0.050	^***^	0.060
		S2	0.503	0.042	^***^	0.253
		S3	0.523	0.041	^***^	0.273
		S4	0.522	0.041	^***^	0.272
		S5	0.882	0.016	^***^	0.778
		S6	0.922	0.019	^***^	0.849
		S7	0.606	0.035	^***^	0.368
Academic resilience in mathematics scale		AR1	0.634	0.043	^***^	0.402
		AR2	0.629	0.043	^***^	0.396
		AR3	0.697	0.041	^***^	0.486
		AR4	0.660	0.042	^***^	0.435
		AR5	0.674	0.039	^***^	0.455
		AR6	0.669	0.049	^***^	0.447
		AR7	0.704	0.045	^***^	0.496
		AR8	0.791	0.030	^***^	0.626
		AR9	0.596	0.051	^***^	0.355

Est, estimate; SE, standard error; R^2^, explained variance. ^***^p < 0.001.

**Table 4:** Confirmatory factor analysis model fit indices for individual measurement models.

Measurement model	χ^2^	df	χ^2^/df	CFI	TLI	RMSEA	SRMR
Social-emotional learning skills scale	259.89	193	1.35	0.913	0.896	0.031	0.068
Math engagement scale	1,457.78	458	3.18	0.909	0.902	0.077	0.079
Academic resilience in mathematics scale	95.43	26	3.67	0.956	0.939	0.085	0.059

Overall, the confirmatory factor analyses indicated acceptable-to-good fit across the measurement models. For the SEL scale, fit indices supported good model fit (χ^2^/df = 1.35, RMSEA = 0.031, CFI = 0.913, TLI = 0.896, SRMR = 0.068). Standardized factor loadings were generally moderate; however, several items showed comparatively low loadings (e.g., SEL21 = 0.190). Notably, items within the Self-Confidence subdimension tended to exhibit lower loadings and *R*^2^ values than items in other subdimensions, suggesting that this subdimension may provide a weaker representation of the higher-order SEL construct in the current sample.

For the mathematics engagement scale, model fit was acceptable (χ^2^/df = 3.18, RMSEA = 0.077, CFI = 0.909, TLI = 0.902, SRMR = 0.079). Most items loaded significantly with moderate-to-high standardized loadings, with particularly strong loadings observed for the Emotional and Social engagement indicators (e.g., ME31 = 0.882; ME32 = 0.922). A small number of items in the Cognitive subdimension showed relatively lower loadings (e.g., ME7 = 0.324), but the overall factor structure remained stable.

For mathematical resilience, the single-factor model demonstrated acceptable fit (χ^2^/df = 3.67, RMSEA = 0.085, CFI = 0.956, TLI = 0.939, SRMR = 0.059). All items loaded significantly on the latent factor, with standardized loadings ranging from 0.596 to 0.791, supporting the unidimensional structure of the scale.

To provide additional evidence for construct reliability and convergent validity, composite reliability (CR) and average variance extracted (AVE) values were calculated for both first-order and second-order latent factors. For the second-order constructs, the overall SEL factor demonstrated good composite reliability (CR = 0.825) with a slightly lower AVE value (AVE = 0.451). Although this AVE estimate was marginally below the conventional 0.50 threshold, the construct was considered to demonstrate acceptable convergent validity, as Fornell and Larcker (1981) noted that convergent validity may still be adequate when AVE is below 0.50 but composite reliability exceeds 0.60. The second-order mathematics engagement construct showed excellent reliability and convergent validity (CR = 0.972, AVE = 0.897). These findings suggest that although some first-order SEL facets were represented weaklier in the present sample, the higher-order SEL construct used in the mediation model remained psychometrically adequate, thereby supporting the validity of the structural analyses.

Taken together, these results indicate that the measurement models showed adequate fit and that the indicators generally captured their intended latent constructs, supporting their use in the subsequent structural analyses.

### 3.3 Assessment of the structural model

Model fit indices for the SEM mediation model are presented in Table 5. The structural model demonstrated acceptable-to-good fit to the data (χ^2^ = 2873.42, df = 1877, χ^2^/df = 1.55, CFI = 0.914, TLI = 0.919, RMSEA = 0.039, SRMR = 0.078). Collectively, these values meet commonly used criteria for acceptable model fit, indicating that the specified mediation model provides a satisfactory representation of the observed covariance structure. The structural model explained 31% of the variance in MR (*R*^2^ = 0.332) and 65% of the variance in ME (*R*^2^ = 0.687).

**Table 5:** SEM-based mediation model fit indices.

Model	χ^2^ (scaled)	Df	χ^2^/df	CFI	TLI	RMSEA	SRMR
SEM-based mediation model	2,873.42	1,877	1,55	0.914	0.919	0.039	0.078

Figure 1 depicts the hypothesized structural relations among the latent constructs: SEL, mathematical resilience, and mathematics engagement. The model was specified to test the direct effect of SEL on engagement as well as the indirect effect via mathematical resilience.

Standardized direct, indirect, and total effects are reported in Table 6. SEL significantly and positively predicted mathematical resilience (path a: β = 0.581, SE = 0.069, *z* = 4.402, *p* < 0.001). Mathematical resilience significantly and positively predicted mathematics engagement (path b: β = 0.591, SE = 0.065, *z* = 7.221, *p* < 0.001). The direct effect of SEL on engagement remained significant (path c′: β = 0.336, SE = 0.072, *z* = 3.380, *p* < 0.001). In addition, the indirect effect of SEL on engagement through resilience was significant (a^*^b: β = 0.343, SE = 0.052, *z* = 4.319, *p* < 0.001). Because the direct pathway (c′) remained significant after accounting for resilience, these findings indicate partial mediation. The total effect of SEL on engagement was significant and comparatively strong (β = 0.679, SE = 0.051, *z* = 4.896, *p* < 0.001), suggesting that SEL is associated with higher engagement both directly and indirectly via mathematical resilience.

**Table 6:** Standardized coefficients for mediation model.

Model	Effect	Path	Estimate	se	*z*	*p*	95% CI Lower	95% CI Upper
SEM-based Mediation Model	Direct effect (a)	SEL → MR	0.581	0.069	4.402	< 0.001	0.440	0.725
	Direct effect (b)	MR → ME	0.591	0.065	7.221	< 0.001	0.434	0.738
	Direct effect (c')	SEL → ME	0.336	0.072	3.380	< 0.001	0.173	0.487
	Indirect effect (a^*^b)	SEL → MR → ME	0.343	0.052	4.319	< 0.001	0.224	0.462
	Total effect	(SEL → MR → ME) + (SEL → ME)	0.679	0.051	4.896	< 0.001	0.571	0.779

Estimates are fully standardized (β). SEL, social and emotional learning; MR, mathematical resilience; ME, mathematics engagement; se, standard error.

## 4 Discussion

The present study examined whether social and emotional learning (SEL) competencies are associated with students' mathematics engagement both directly and indirectly through mathematical resilience (MR) during early adolescence. The results revealed a strong total association between SEL and mathematics engagement (β = 0.679), indicating that socio-emotional competencies are meaningfully associated with students' participation in mathematical learning. By examining the SEL → MR → Engagement pathway, the study offers insights into how broad socio-emotional competencies may translate into domain-specific adaptive dispositions that are associated with sustained engagement in mathematics. The findings further indicate a partial mediation structure in which SEL is associated with engagement through two complementary mechanisms: a resilience pathway that is linked to persistence during difficulty and a direct pathway that is associated with participation in the relational and communicative practices of mathematics classrooms.

### 4.1 SEL as a predictor of mathematical resilience

Consistent with Hypothesis 1, SEL significantly predicted mathematical resilience (β = 0.581), indicating that socio-emotional competencies are meaningfully associated with shaping how students respond to the cognitive and emotional demands of mathematics. Mathematics learning frequently involves uncertainty, frustration, and the possibility of error (Morkoyunlu and Ayhanöz, 2021; Lee and Ward-Penny, 2022). When such experiences are not effectively regulated, they can escalate into mathematics anxiety, which interferes with working memory and problem-solving performance (Ashcraft and Krause, 2007; Ramirez et al., 2018; Barroso et al., 2021). Under these conditions, students may interpret difficulty as evidence of inability rather than as a normal component of learning, reducing persistence and engagement (Ganley and McGraw, 2016; Luttenberger et al., 2018; Skaalvik, 2018).

Mathematical resilience provides a domain-specific framework for understanding how students respond to these challenges. Defined as the capacity to remain productively engaged despite difficulty while maintaining curiosity, confidence, and constructive learning beliefs (Johnston-Wilder and Lee, 2010), MR includes valuing mathematics, normalizing struggle, maintaining growth-oriented beliefs, overcoming learned helplessness, and seeking support when necessary (Lee and Johnston-Wilder, 2014; Kooken et al., 2015). These dispositions rely on emotional regulation, persistence, and relational competencies that are central to contemporary SEL frameworks (CASEL, 2025; OECD, 2021; WHO, 1994). From this perspective, mathematical resilience can be interpreted as a domain-specific manifestation of broader socio-emotional competencies.

The mechanisms linking SEL to MR appear to operate across multiple levels of learning processes. At the intrapersonal level, SEL is associated with emotional regulation, growth-oriented beliefs, and metacognitive monitoring during mathematical activity (Eriksen et al., 2025; Gidalevich and Mirkin, 2024). These capacities help students interpret confusion and error as manageable aspects of learning rather than as threats to competence. At the interpersonal level, SEL is linked to stronger relationship skills that facilitate help-seeking, dialogue, and collaborative reasoning, which are central processes in sociocultural models of learning (Vygotsky, 1978; Lee and Johnston-Wilder, 2017). At the sociocultural level, SEL contributes to a sense of belonging and psychological safety within learning environments, enabling students to remain engaged in collaborative mathematical activity (Lee, 2022).

This interpretation is further supported by empirical evidence showing that students with stronger SEL skills demonstrate greater emotional resilience, self-efficacy, and capacity to cope with academic challenge (Turan, 2021; Min et al., 2024), and that mathematical resilience is consistently associated with higher emotional intelligence and intrinsic motivation in mathematics (Faradillah and Wulandari, 2021; Ogoy and Comahig, 2025).

### 4.2 Academic resilience in maths as a driver of engagement

Supporting Hypothesis 2, mathematical resilience strongly predicted mathematics engagement (β = 0.591). This finding aligns with motivational systems theories that conceptualize engagement as the behavioral, emotional, cognitive, and social expression of students' self-system processes (Connell and Wellborn, 1991; Rimm-Kaufman et al., 2015; Skinner et al., 2009). Students with higher levels of MR tend to perceive difficulty as manageable and meaningful, and this orientation is associated with greater engagement across multiple dimensions of learning.

At the behavioral and cognitive levels, resilient learners tend to persist through difficulty, sustain attention, and reattempt challenging problems rather than withdrawing. These patterns are supported by perseverance, strategic help-seeking, and flexible strategy use (Kooken et al., 2015; Papageorgiou et al., 2025). Cognitively, MR encourages deeper processing and metacognitive monitoring, allowing students to regulate their problem-solving strategies and maintain focus on complex tasks (Cleary and Zimmerman, 2012; Greene, 2015). Because resilient students interpret confusion as a natural component of learning rather than as evidence of inability, they are more likely to invest sustained effort in understanding mathematical problems.

Emotionally, MR is associated with greater regulatory capacity. Students higher in MR tend to manage frustration more effectively and reinterpret errors as informative feedback, which is in turn associated with greater interest, enjoyment, and sustained focus during challenging mathematical activity (Morkoyunlu and Ayhanöz, 2021; Rimm-Kaufman et al., 2025). Emotional regulation therefore represents an essential condition for maintaining engagement when students encounter difficulty (Wang et al., 2016).

MR also appears to support participation in the social practices of mathematics learning. Contemporary mathematics instruction increasingly emphasizes explanation, discourse, and collaborative reasoning (Wang et al., 2016; Wang and Eccles, 2013). Participating in such practices requires students to articulate emerging ideas, consider alternative perspectives, and engage with peer feedback. Students with higher levels of mathematical resilience may therefore be more willing to take interpersonal risks during collaborative reasoning and contribute actively to shared problem solving. In this sense, MR is associated not only with individual persistence but also with participation in the social practices of mathematical learning communities (Lave and Wenger, 1991).

These patterns are consistent with recent empirical findings: mathematical resilience has been found to correlate strongly with student engagement in mathematics (*r* = 0.716) among secondary students, and to partially mediate the relationship between mathematics self-efficacy and engagement (Digamo and Abuzo, 2024). Longitudinal evidence similarly shows that resilience is positively associated with online mathematics learning engagement over time (Liu, 2024), while a systematic review confirms that mathematically resilient learners persistently engage more actively in mathematical activity (Xenofontos and Mouroutsou, 2022).

### 4.3 Integrating the model: The dual pathway from SEL to mathematics engagement

Consistent with Hypotheses 3 and 4, SEL was associated with mathematics engagement both directly (β = 0.336, *p* < 0.001) and indirectly through mathematical resilience (indirect effect = 0.343, *p* < 0.001). The total association between SEL and mathematics engagement was strong (β = 0.679, *p* < 0.001), and the overall model explained a substantial proportion of variance in mathematics engagement (*R*^2^ = 0.69). Given the modest internal consistency of the retained SEL measurement model in this sample, however, the SEL-related direct and indirect associations should be interpreted with appropriate caution.

The direct pathway highlights the role of SEL competencies in supporting students' readiness to participate in mathematics classrooms. Mathematics learning often involves explaining reasoning, engaging in discussion, and collaborating with peers during cognitively demanding activity (Tandika et al., 2025). These practices require not only cognitive resources but also socio-emotional capacities that support communication, cooperation, and self-regulation (Jensen, 2024). SEL competencies may therefore directly predict engagement, as students with stronger SEL skills are better positioned to participate in the relational and communicative processes through which mathematical understanding develops.

Empirical research supports this interpretation. A systematic review of 91 studies found that social and emotional competencies are positively associated with academic engagement across educational levels (Santos et al., 2023). SEL competencies have further been associated with behavioral, emotional, and cognitive engagement as well as with improved classroom participation and peer interaction (Immordino-Yang et al., 2019; Divecha and Brackett, 2020). In mathematics education contexts, SEL-oriented instructional practices have similarly been linked to greater participation, cooperation, and persistence in mathematical activity (Molyneux and Diamond, 2025; Eriksen et al., 2025; Karacan, 2022; Bhoumick and Saha, 2020).

The indirect pathway through mathematical resilience reflects a complementary mechanism that becomes particularly important when students encounter difficulty. Mathematics learning frequently involves uncertainty and the possibility of failure, experiences that can trigger anxiety and avoidance when students lack sufficient emotional resources (Johnston-Wilder and Lee, 2010; Lee and Johnston-Wilder, 2014). Within such contexts, students who have developed stronger mathematical resilience through their SEL competencies are better equipped to remain engaged rather than withdrawing —thus the indirect pathway becomes particularly consequential under conditions of difficulty.

One proposed associative pathway linking SEL to resilience involves emotional regulation. Mathematics learning often evokes frustration and uncertainty (Boaler, 2019; Sears et al., 2022). Students with stronger socio-emotional competencies may be better able to regulate these responses and interpret difficulty as a manageable aspect of learning. Control-Value Theory (Pekrun, 2006) provides a useful framework for understanding this process, suggesting that achievement emotions depend on students' perceived control and value within learning situations. Students with stronger SEL competencies—and thus greater emotional regulation and perceived control—may therefore be more likely to remain engaged when encountering challenging mathematical tasks.

Recent empirical findings further reinforce this interpretation. Aaviste et al. (2026) report that emotional control, stress resistance, and perseverance are among the strongest predictors of adaptive mathematics functioning and lower mathematics anxiety. These competencies correspond closely to the regulatory and persistence-related components of SEL that appear relevant to mathematical resilience. Within the present model, mathematical resilience may therefore be interpreted as a domain-specific construct through which broader socio-emotional resources are associated with sustained engagement under conditions of academic challenge. This interpretation is also consistent with recent evidence showing that mathematical resilience partially mediates the relationship between mathematics self-efficacy and student engagement (Digamo and Abuzo, 2024).

The present study's measurement findings raise an additional, but necessarily cautious, interpretive issue. The SEL-Persistence dimension was removed from the measurement model due to a Heywood case, indicating an inadmissible parameter estimate. However, Heywood cases may arise from several sources, including model specification, item structure, high factor correlations, sample characteristics, or other technical features of the measurement model (Cooperman and Waller, 2022; Kolenikov and Bollen, 2012). Therefore, this result should not be interpreted as definitive evidence regarding the theoretical status of Persistence. Nevertheless, given the conceptual proximity between persistence and mathematical resilience, the finding may point to a possible overlap between domain-general SEL persistence and mathematics-specific resilience. Future studies should examine whether persistence is best represented as a distinct SEL competency, as part of mathematical resilience, or as a construct that bridges both domains.

The present findings may also be interpreted in relation to evidence emerging from the Turkish educational context. In their qualitative investigation of students' engagement in school, Kina and Karacan Ozdemir (2026) reported that teachers and parents frequently associated low academic engagement with students' lack of interest in lessons, reluctance to attend school, and limited sense of purpose regarding schooling. Importantly, participants in the same study also identified deficiencies in key social–emotional competencies—such as self-awareness, self-management, social awareness, relationship skills, and responsible decision-making—as central barriers to students' active participation in learning. The overlap between the barriers to engagement identified by practitioners and the mechanisms identified in the present study provides convergent support for the role of socio-emotional competencies in shaping students' participation in learning processes.

These findings are also consistent with recent educational developments in Türkiye that emphasize holistic and skill-based approaches to learning. Within the K-12 Skills Framework Türkiye Integrated Model, learning is conceptualized as the interaction of conceptual skills, domain-specific skills, and social–emotional competencies that together support the development of domain competence (Aşkar and Altun, 2023; Karabey and Erdogan, 2023). In this perspective, mathematical competence extends beyond knowledge and procedural skills to include dispositions, emotional regulation, and adaptive responses to mathematical challenges. The dual pathway identified in the present study therefore aligns with this integrated framework, suggesting that strengthening students' socio-emotional competencies may represent an important pathway for sustaining engagement with cognitively demanding mathematical activity.

## 5 Conclusion and implications

This study examined the role of social and emotional learning (SEL) competencies in students' mathematics engagement during early adolescence. The findings indicate that SEL is associated with mathematics engagement both directly and indirectly through mathematical resilience. This pattern suggests that socio-emotional competencies are linked to engagement not only through students' participation in classroom interaction but also through adaptive responses to mathematical difficulty. In this sense, mathematical resilience represents an important associative pathway through which broader socio-emotional competencies relate to sustained engagement with mathematics learning.

The results contribute to research emphasizing the interconnected nature of cognitive, emotional, and social processes in academic engagement. The identification of mathematical resilience as a mediating mechanism suggests how socio-emotional competencies are associated with both participation in mathematical activity and persistence when students encounter challenge.

### 5.1 Educational implications

For classroom teachers, the findings suggest that mathematics instruction may benefit from practices that simultaneously address cognitive and socio-emotional demands. Specifically, normalizing productive struggle—through explicit framing of errors as learning opportunities and encouragement of help-seeking—may strengthen mathematical resilience alongside content learning. Teachers might also incorporate structured collaborative tasks that require students to articulate reasoning, negotiate meaning, and provide peer feedback, thereby engaging both the cognitive and relational dimensions of mathematics engagement. However, effective implementation requires distinguishing between declarative knowledge of SEL competencies and their procedural enactment within authentic classroom routines; students are unlikely to develop emotional regulation or growth-oriented beliefs through awareness alone without guided practice and real-time modeling (Alemdar et al., 2026).

Notably, the particularly high loadings observed for social engagement indicators in the present study (e.g., DK32 = 0.922) suggest that peer-relational experiences are especially salient in mathematics classrooms. Although the present study did not directly test specific instructional techniques, the findings are compatible with the view that mathematics engagement is shaped partly by social interaction, peer relations, and communicative participation. Accordingly, cooperative learning structures such as Student Teams–Achievement Divisions (STAD), Jigsaw, and Think-Pair-Share may offer useful contexts for fostering peer explanation, shared reasoning, and relational participation in mathematics classrooms. Cooperative learning, when structured around principles such as positive interdependence, individual accountability, and face-to-face interaction (Johnson and Johnson, 1999), may provide students with opportunities to explain their reasoning, negotiate mathematical meaning, and build on one another's ideas in small groups. However, these practices should be understood as pedagogical approaches that are theoretically aligned with the present findings, rather than as interventions whose effectiveness was directly demonstrated in this study. Future experimental or classroom-based intervention studies are needed to examine whether such approaches strengthen SEL-related engagement processes in mathematics learning.

For policymakers, the present findings provide empirical support for the K-12 Skills Framework Türkiye Integrated Model's positioning of socio-emotional competencies as foundational to domain-specific learning. The results suggest that SEL provision should not be conceptualized as separate from or competing with mathematics education, but as structurally embedded within it. In curriculum-driven systems where academic priorities tend to dominate classroom time, conceptualizing SEL as a set of transferable core practices or “kernels” integrated into existing mathematics routines may offer a pragmatic and scalable pathway (Jones et al., 2017). Sustained teacher professional development that strengthens teachers' own socio-emotional competencies, pedagogical strategies, and reflective practices is a prerequisite for this integration to be effective rather than merely nominal.

For curriculum developers, the dual-pathway model identified in this study suggests that mathematics curricula should attend to both the resilience-building and relational dimensions of engagement. Curriculum materials that embed prompts for metacognitive reflection, growth-oriented self-assessment, and collaborative mathematical reasoning may help activate both pathways identified in the present model. Whole-school coherence, ensuring alignment between classroom practices, school culture, and family engagement, is also important, as SEL competencies develop through relational continuity rather than discrete instructional episodes.

### 5.2 Limitations and future directions

The present study has several limitations that should be considered when interpreting the findings. First, the correlational design precludes causal inference; all observed associations should be interpreted as directional hypotheses requiring experimental or longitudinal validation rather than as evidence of causal mechanisms.

Second, because all variables were measured through student self-report instruments collected at a single time point, the observed associations may be partially influenced by common method variance. Although the structural model demonstrated good fit, future studies should consider multi-informant designs incorporating teacher ratings or behavioral observations, or longitudinal data collected across multiple waves, to reduce shared method bias and better establish the temporal ordering of the constructs.

Third, because participants were recruited through convenience sampling from three public schools in a single metropolitan area, the findings may not generalize directly to middle school students in different regions, rural or private school settings, or socioeconomically diverse educational contexts. Replication with probability-based and geographically diverse samples is warranted before broader conclusions are drawn.

Fourth, some measurement-related limitations should be acknowledged. The reliability concern refers specifically to the estimates obtained in the present sample and retained measurement model, rather than to the psychometric adequacy of the original SEL scale. In this study, the retained SEL measurement model yielded relatively modest internal consistency estimates. Because SEL was the main predictor in the structural model, this may have reduced the precision of the estimated paths involving SEL and may have attenuated the observed direct and indirect associations. In addition, several items and the SEL-Persistence dimension were removed during analysis due to psychometric concerns. Although these decisions were methodologically justified, they may have narrowed the representation of the SEL construct and may limit direct comparability with studies using the full original scale. Future studies should re-examine the dimensional structure of SEL in similar populations and provide additional validity evidence using alternative measurement approaches.

Notwithstanding these limitations, the present study makes a meaningful contribution by providing the first empirical test of a dual-pathway model linking SEL competencies to mathematics engagement through mathematical resilience among middle school students. Future research may build on these findings by employing longitudinal or mixed-method designs to examine how SEL competencies, mathematical resilience, and engagement develop and interact over time and across diverse learning contexts.

## Data Availability

The datasets presented in this study can be found in online repositories. The names of the repository/repositories and accession number(s) can be found below: https://osf.io/859xg.
